# When snouted wild-boars routing tender corn / Anger our huntsman^1^

**DOI:** 10.3201/eid1712.AC1712

**Published:** 2011-12

**Authors:** Polyxeni Potter

**Affiliations:** Centers for Disease Control and Prevention, Atlanta, Georgia, USA.

**Keywords:** art science connection, emerging infectious diseases, art and medicine, Jan Fyt, Atalanta and Meleager Hunt the Calydonian Boar, When snouted wild-boars routing tender corn, Anger our huntsman, animaliers, pigs, zoonoses

**Figure:**
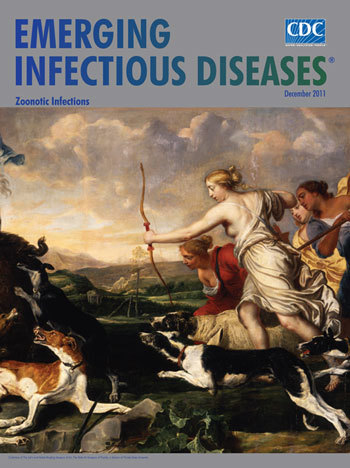
**Jan Fyt and others (1611–1661) *Atalanta and Meleager Hunt the Calydonian Boar* (1648) Oil on canvas (243.8 cm × 411.5 cm). **Bequest of John Ringling, 1936, Collection of The John and Mable Ringling Museum of Art http://www.ringling.org/, The State Art Museum of Florida, a division of Florida State University

“A monster sent by the gods to ravage the vineyards” is how the Calydonian boar was described in mythologic accounts. King Oeneus did not offer proper thanks to Artemis, goddess of the hunt. In retaliation, she unleashed the monster on the fertile fields of Calydon, “an ornament to Greece,” terrorizing adjacent communities and wreaking havoc with local agriculture. “Now it trampled the young shoots of the growing crops, now cut short the ripeness, longed-for by the mournful farmer, and scythed down the corn in ear,” wrote Ovid in his Metamorphoses. “Its tusks were the size of an Indian elephant’s: lightning came from its mouth: and the leaves were scorched by its breath.”

Oeneus’ son, Meleager assembled a team of braves to go after the beast. Among these were Jason, leader of the Argonauts; Theseus king of Athens; and swift-footed Atalanta, “the warrior girl of Tegea,” a creature of the forest, suckled by a she-bear after being abandoned by her father, who had wished for a son.

The boar, “flame burning in its eyes,” charged the hunters “like lightening forced from colliding clouds,” killing several before it was finally wounded by Atalanta’s arrow and finished off by Meleager who, impressed by the heroine’s performance, offered her the pelt as trophy. Meleager’s gesture offended the team. In the scuffle that followed, he killed two of his uncles. His mother, distraught by the loss, allowed her son’s death to avenge them.

The story of the Calydonian boar hunt was mentioned in The Iliad and was retold many times, in greatest detail by Ovid. “The boar was furiously twisting its body round and round, its jaws slavering with foam and fresh blood …. The hero who had dealt the wound came up close to the animal and roused his foe to fury, before finally burying his shining spear in its shoulder.”

Member of the species *Sus scrofa*, family Suidae, and ancestor of the domestic pig, the wild boar roamed much of the world. Native to Europe and the Mediterranean region, the large-bodied, short-legged, coarse and bristled beast, was one of the first to be tamed and live among humans, featured in the mythology of many nations: Egypt, Persia, China, New Guinea, India. In the wild, it embodied, alongside a penchant for savage behavior, other, much coveted, virtues: power, courage, nobility, cleverness, initiative. In the hierarchy of wild animals, it was right up there, just below the lion, followed by birds of prey.

Legends we read for their timelessness and to understand the world and its complexities, ancient and modern: angry gods, fearless heroes, family strife, the female athlete, men and women, and no less the epic combat between humans and animals, their common destiny, and their close evolutionary and spiritual connection. The Atalanta tale from classical antiquity has had many interpretations. One, most persistent, concerns the relationship between nature and culture—animals and their behavior in the wild, humans and their behavior in societies, and the inevitable link between them.

Artists have long drawn inspiration from legends, and the Calydonian boar hunt was depicted often, as early as 2,500 years ago by the potter Ergotimos and the painter Kleitias, and throughout art history. Peter Paul Rubens, an avid painter of historical and mythologic themes, created his own version. Another famous presentation is Jan Fyt’s effort on this month’s cover. Fyt, a native of Antwerp, who trained with accomplished still life painter and animalier Frans Snyder, traveled to Italy and France but returned to live and work in his home town. A draftsman and etcher as well as painter, he excelled in the depiction of animals, which he presented with flourish in hunting scenes or within compositions filled with exquisite porcelain, fabrics, flowers, and fruit. At times he collaborated with other artists, among them Willeboirts Bosschaert and Jacob Jordaens.

In his version of the myth, Fyt captured with the brush the fierceness of the battle as effectively as Ovid did in words. “There was a deep valley … and it held in its depths pliant willows, smooth sedges, and marsh grasses.” The swift arrow from the girl from Tegea “grazed the top of the boar’s back, and fixing itself below one ear, reddened the bristles with a thin stream of blood.” The braves circled as the hunting dogs, dwarfed by the massive beast, lay injured and worse.

The symbolic nature of the myth lies in the struggle between the wild animal and the human community, with the beast destabilizing the social fabric and triggering mayhem, much to the detriment of both. Animal and human communities have undergone massive changes since the crisis in Calydon. Although wild boars still occasionally tear up rural villages, they pose scarcely a risk to urban areas, which themselves hardly resemble the mythologic vineyards. Yet the struggle persists and not only on the level of the hunt but also on the microbiologic level, come to the surface far too late for mythologic coverage but filled with intrigue nonetheless.

Instead of wild boars plundering the countryside, we now have in much of the world domesticated swine herds, although in developing countries, pigs are often allowed to roam in the community, where they no longer terrorize the populace with oversized tusks but cause other problems. In the domesticated setting, pigs and humans share commensal organisms in the gut that can cause extraintestinal disease when opportunities arise, as in the health care setting. But often, where there is disease in pigs, there is also disease in humans.

With some zoonotic infections, such as in this issue trichinellosis and swine brucellosis, infected pigs continue to be a threat. *Streptococcus suis*, which causes meningitis and septicemia in piglets and occupational disease in humans, is reported to be a cause of adult streptococcal infection in Vietnam and Thailand and of outbreaks in the People’s Republic of China. Humans and pigs can harbor clinical *Enterococcus faecalis.* A link of two intestinal commensal porcine-origin *E. faecalis* strains in Denmark strengthens evidence that pigs can be a source of these infections in humans.

While contact with our wild counterparts will continue to result in emerging human disease, effective measures have limited exposure to many zoonotic agents in regions with strong programs in domestic animal disease control. But until control of livestock infections is universally used to safeguard public health, these infections will continue to pose a threat to humans and their domesticated animal partners around the world. Or as Keats predicted in “Endymion,” “Again my trooping hounds their tongues shall loll / Around the breathed boar.”
